# Tomato transgenic plants expressing hairpin construct of a nematode protease gene conferred enhanced resistance to root-knot nematodes

**DOI:** 10.3389/fmicb.2015.00260

**Published:** 2015-04-01

**Authors:** Tushar K. Dutta, Pradeep K. Papolu, Prakash Banakar, Divya Choudhary, Anil Sirohi, Uma Rao

**Affiliations:** Division of Nematology, ICAR-Indian Agricultural Research InstituteNew Delhi, India

**Keywords:** root-knot nematode, *Mi-cpl-1*, RNAi, transgenics, resistance

## Abstract

Root-knot nematodes (*Meloidogyne incognita*) cause substantial yield losses in vegetables worldwide, and are difficult to manage. Continuous withdrawal of environmentally-harmful nematicides from the global market warrants the need for novel nematode management strategies. Utility of host-delivered RNAi has been demonstrated in several plants (Arabidopsis, tobacco, and soybean) that exhibited resistance against root-knot and cyst nematodes. Herein, a *M. incognita*-specific protease gene, cathepsin L cysteine proteinase (*Mi-cpl-1*), was targeted to generate tomato transgenic lines to evaluate the genetically modified nematode resistance. *In vitro* knockdown of *Mi-cpl-1* gene led to the reduced attraction and penetration of *M. incognita* in tomato, suggesting the involvement of *Mi-cpl-1* in nematode parasitism. Transgenic expression of the RNAi construct of *Mi-cpl-1* gene resulted in 60–80% reduction in infection and multiplication of *M. incognita* in tomato. Evidence for *in vitro* and *in vivo* silencing of *Mi-cpl-1* was confirmed by expression analysis using quantitative PCR. Our study demonstrates that *Mi-cpl-1* plays crucial role during plant-nematode interaction and plant-mediated downregulation of this gene elicits detrimental effect on *M. incognita* development, reinforcing the potential of RNAi technology for management of phytonematodes in crop plants.

## Introduction

Root-knot nematodes (*Meloidogyne* spp.) are one of the potential constraints for cultivation of vegetables particularly in the tropical and subtropical countries. In India, *Meloidogyne incognita* race 1 is most prevalent causing considerable damage especially in solanaceous and cucurbit crops (Jain et al., 2007). The infective second stage juvenile (J2) penetrates at the root tip zone and migrates intercellularly until it reaches the differentiating vascular cylinder. During invasion, nematodes inject a cascade of effector proteins of esophageal gland origin into plant cells via its stylet (Hassan et al., 2010). These effectors are thought to be involved in host pathogen interaction starting from the host recognition process to degradation of plant cell walls in order to facilitate the migration of nematode, culminating in the establishment of hypermetabolic, multinucleate feeding cell (giant cell, GC) which serves as the permanent food source for nematode development and reproduction (Davis et al., 2008). Cortical cells surrounding the GC are proliferated through hyperplasia to form the gall. Due to the formation of knots or galls around the feeding site in vascular tissue, upward translocation of water and nutrient in the root is affected, resulting in the reduction of crop yield (Moens et al., 2009).

Existing management practices such as the use of nematicides are posing a threat on the environment and are costly. Therefore, resorting to the environmentally benign and cost-effective nematode management strategies is the preferred alternative. In the recent years, RNA interference (RNAi) has emerged as a potential tool to manage the crop pathogens through host-induced gene silencing (HIGS) approach (Koch and Kogel, 2014). Plethora of nematode genes was knocked down using HIGS approach, causing reduction in parasitic success of root-knot and cyst nematodes in different crop plants (Dutta et al., 2015). Due to its precise selectivity for the target organism with least side effects, RNAi can be utilized as a remarkable tool to develop nematode resistant transgenic plants.

Proteinases are ubiquitous proteolytic enzymes that cleave the internal peptide bonds within proteins and peptides, found in a wide range of organisms such as bacteria, plants, invertebrates and vertebrates. In case of parasitic helminths, papain superfamily of cysteine proteinases (i.e., cathepsins) has drawn the most attention (Tort et al., 1999). Based on the presence and absence of a distinctive set of amino acids within the polypeptide, phylogenetic analysis identified more than 10 subdivisions within the cathepsin superfamily including cathepsin B, C, L, and Z, among which cathepsin L and Z-like proteases are exclusively present in many parasitic nematodes, and have potential roles in invasion and feeding on host tissues, molting, and evasion of innate host defenses (Santamaria et al., 1998; Koiwa et al., 2000; Shompole and Jasmer, 2001).

Among the plant-parasitic species, cysteine proteinase activity was detected in potato cyst nematode (*Globodera pallida*), while cathepsin like cysteine proteinase activity was identified in the intestine of feeding females of soybean cyst nematode, *Heterodera glycines* (Lilley et al., 1996). Neveu et al. (2003) characterized a cathepsin L protease full length cDNA (*Mi-cpl-1*) from infective J2 of *M. incognita*, expression of which was shown to occur exclusively in the developmental stages (i.e., J2 and females), indicating the possible role of *Mi-cpl-1* in nematode development. Transcripts of the *Mi-cpl-1* accumulated specifically in the intestinal cells of nematodes, suggesting their involvement in the digestive function of nematodes.

Induction of RNAi upon ingestion of double-stranded RNA (dsRNA) was proved to be effective in the free-living nematode, *Caenorhabditis elegans*, during *in vitro* experiments (Fire et al., 1998). Subsequently, cathepsin L-like cysteine proteinases were used as the target gene in a number of *in vitro* RNAi studies. According to Hashmi et al. (2002), RNAi of *Ce-cpl-1*, a *C. elegans* cathepsin L protease, resulted in embryonic lethality and delayed the growth of larvae to egg producing adults, indicating the activity of *Ce-cpl-1* is correlated with the embryogenesis and post-embryonic development process of *C. elegans*. RNAi of cysteine protease genes (*falcipain-1* and -*2*) caused severe morphological abnormalities in the malaria parasite, *Plasmodium falciparum* (Malhotra et al., 2002). In case of human filarial parasite (*Onchocerca volvulus*), RNAi of two specific genes, i.e., cathepsin L (*Ov*-*cpl*) and cathepsin Z (*Ov*-*cpz*) inhibited the molting of third stage (L3) to fourth stage larvae (L4) (Lustigman et al., 2004).

For the functional validation of cysteine proteinase genes in plant nematodes, dsRNA delivery was accomplished by soaking the nematodes with dsRNA solution mixed with the neurotransmitters like resorcinol, octopamine, serotonin etc. Silencing of the cysteine proteinases of *H. glycines* (*hgcp-I*) and *G. pallida* (*gpcp-I*) led to the altered sexual fate and less recovery of egg laying females (Urwin et al., 2002). A reduction in the transcript abundance along with the reduced infectivity was observed while *in vitro* RNAi was used to investigate the function of *Mi-cpl-1* gene in *M. incognita*. Downregulation of *Mi-cpl-1* gene led to the reduced parasitic success of *M. incognita* (Shingles et al., 2007).

Despite the reports of *in vitro* studies, cysteine proteinase genes have not yet been extensively targeted for the HIGS approach. In an isolated report, tobacco transgenic lines expressing dsRNA for *Mi-cpl-1* gene imparted partial resistance to *M. incognita* race 3 (Antonino de Souza Júnior et al., 2013). In the current investigation, *Mi-cpl-1* gene was knocked down using *in vitro* as well as *in planta* RNAi approach to analyse the function of this important protease in plant-nematode interaction. Tomato transgenic lines were generated which exhibited resistance to *M. incognita* race 1. In addition to that, single copy transgenic events were generated and target gene small RNAs were detected in the transgenic roots, through Southern and northern analysis, respectively. Reduction in the transcript level of *Mi-cpl-1* was detected in the females that developed in the transgenic plants.

## Materials and methods

### Culturing of nematodes

A pure culture of *M. incognita* (Kofoid & White) Chitwood race 1 was maintained on tomato (*Solanum lycopersicum* L. cv. Pusa Ruby) in a glasshouse. Egg masses were collected from the roots of 2 months old plant using sterilized forceps and were kept for hatching in a double-layered paper tissue supported on a molded sieve of wire gauze in a Petri dish containing distilled water (Hooper, 1986). Freshly hatched J2s were used for all the experiments.

### Selection of target region of *Mi-cpl-1* gene

**Figure 1:**
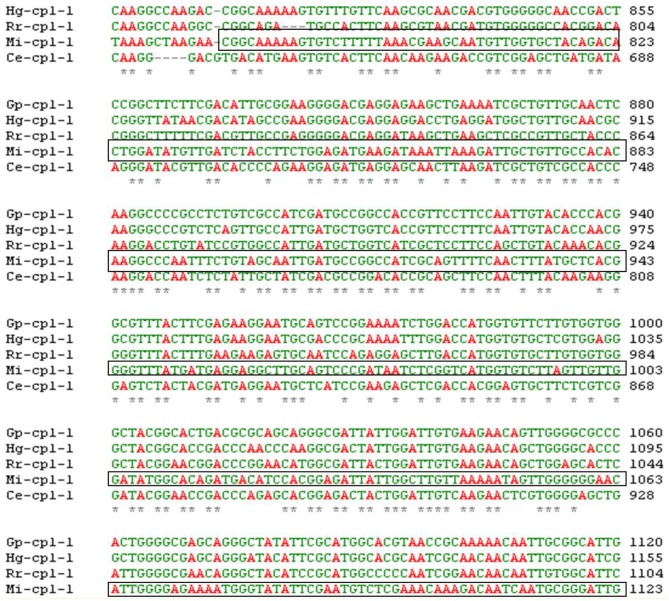
**Multiple sequence alignment of *Mi-Cpl-1* gene with the homologous gene sequences of different nematode species**. *Shows the conserved region across the nematode species. Boxed portion indicates the target region of *Mi-cpl-1* for RNAi studies. GenBank ID for *Mi-Cpl-1* (1152 bp), *Gp-Cpl-1* (1305 bp), *Hg-Cpl-1* (1356 bp), *Rr-Cpl-1* (1264 bp) and *Ce-Cpl-1* (1192 bp) are AJ557572.1, AY999065.1, AY554271.1, AY999066.1, and NM_074798, respectively.

A pairwise alignment of full length sequence of *Mi-cpl-1* showed 66, 68, 63, and 59% identity with the other homologous gene sequences including *Gp-cpl-1* of *G. pallida, Hg-cpl-1* of *H. glycines, Rr-cpl-1* of *Rotylenchulus reniformis* and *Ce-cpl-1* of *C. elegans*, respectively, at the nucleotide level using ClustalW programme (Larkin et al., 2007). The probability of silencing is reduced if the two related genes are <90% homologous (Sharp, 2001) and even a single nucleotide mismatch between a siRNA molecule and its target mRNA may prevent the RNAi process (Elbashir et al., 2001). Therefore, silencing of *Mi-cpl-1* may not induce any off-target effects on non-target organisms. A 366 bp sequence (spanning between 787 and 1152 bp of *Mi-cpl-1* gene) conserved across the similar gene family of other nematode species, was identified as the target sequence for RNAi construct development (Figure 1).

### Cloning and sequencing of *Mi-cpl-1* gene

Total RNA was isolated from J2s using Trizol reagent (Invitrogen) and digested with DNaseI (New England Biolabs) to remove the contaminating DNA molecules. The quantity and quality of the extracted RNA molecule were assessed using Nanodrop ND-1000 spectrophotometer (Thermo Scientific). Approximately 400 ng of purified RNA was reverse transcribed to cDNA using cDNA synthesis Kit (Superscript VILO, Invitrogen). A 366 bp region of *Mi-cpl-1* (GenBank ID: AJ557572) was amplified from the cDNA using the primers as given in Table 1. The fresh PCR product was cloned into pGEM-T Easy (Promega, USA) using standard protocol and transformed into *Escherichia coli* DH5α competent cells (New England Biolabs). The plasmid was recovered from positive colonies (QIAGEN Plasmid Miniprep kit) and sequenced (ABI SOLiD sequencing platform) to ascertain the identity of the insert.

**Table 1:** **List of primers and probes used for cloning, PCR amplification, Southern and northern hybridization**.

**Probe/gene**	**Primer name**	**Primer sequence (5′-3′)**	**Product length (bp)**	**Tm (°C)**
**PCR AMPLIFICATION, SOUTHERN AND NORTHERN ANALYSIS**				
Mi-cpl-1 (AJ557572)	Mi-cpl-1F	TGTCTTTTTAAACGAAGCAA	366	60
	Mi-cpl-1R	TTAAACAAGTGGATATGAAG		
U6 (X60506)	U6F	GCGCAAGGATGACACGCA	439	60
	U6R	GGCTGAGTTATTTTTTTCTG		
**CLONING OF *Mi-cpl-1* IN RNAi VECTOR, GENOTYPING OF TRANSGENIC PLANTS**				
Mi-cpl-1 attB	Mi-cpl-1_attB F	^*^(AttB1)-TGTCTTTTTAAACGAAGCAA	424	60
	Mi-cpl-1_attB R	^*^(AttB2)-TTAAACAAGTGGATATGAAG		
CaMV35S promoter	35S F	TCCTTCGCAAGACCCTTC		
OCS terminator	OCS F	CTTCTTCGTCTTACACATCACTTGTC		
nptII	nptII F	CAA TCG GCTGCTCTCATGCCG	750	60
	nptII R	AGGCGATAGAAGGCGATGCGC		
**EXPRESSION ANALYSIS**				
Mi-cpl-1 (AJ557572)	Mi-cpl-1_RT F	CTACCTTCTGGAGATGAAGATAAA	129	60
	Mi-cpl-1_RT R	GCAAGCCTCCTCATCATAAA		
18S rRNA (*S. lycopersicum*) (X51576)	18S_Sl_RT F	CGCGCGCTACACTGATGTATTCAA	172	60
	18S_Sl_RT R	TACAAAGGGCAGGGACGTAGTCAA		
18S rRNA (*M. incognita*) (HE667742)	18S_Mi_RT F	TCAACGTGCTTGTCCTACCCTGAA	155	60
	18S_Mi_RT R	TGTGTACAAAGGGCAGGGACGTAA		
GFP (HF675000)	*gfp* RT F	AGCGGCACGACTTCTTCA	750	60
	*gfp* RT R	GTGTGGACAGGTAATGGTTGT		

* *(AttB1): GGGGACAAGTTTGTACAAAAAAGCAGGCT*.

* *(AttB2): GGGGACCACTTTGTACAAGAAAGCTGGGT*.

### Comparative expression of *Mi-cpl-1* in different life stages of *M. incognita*

To quantify the expression of *Mi-cpl-1* throughout the life cycle of *M. incognita*, quantitative real-time PCR (qRT-PCR) was performed with cDNA from different developmental stages (eggs, J2s and mature females). Total RNA was isolated and synthesized to cDNA as explained above. qRT-PCR was performed in a realplex^2^ thermal cycler (Eppendorf) using SYBR Green Supermix kit (Eurogentec). Reaction mixture for each sample contained a final volume of 10 μl, containing 5 μl of SYBR Green PCR Master mix (Eurogentec), 750 nM of each primer and 1.5 ng of cDNA. To normalize the gene expression level *18S rRNA*, a constitutively expressed gene was used as the reference. The amplification reactions were run using the following programme: a hot start of 95°C for 5 min, followed by 40 cycles of 95°C for 15 s and 60°C for 1 min. After 40 cycles a melt curve analysis or dissociation programme (95°C for 15 s, 60°C for 15 s, followed by a slow ramp from 60°C to 95°C) was acquired to ensure the specificity of amplification. At least two biological and three technical replicates were used for each of the samples. After obtaining the Ct values the 2^−ΔΔCT^ method was used to quantify the relative fold change in gene expression (Livak and Schmittgen, 2001), and student's *t*-test was performed. Primer details are given in Table 1.

### Preparation of dsRNA for *in vitro* RNAi

*Mi-cpl-1* gene was PCR amplified from the pGEM-T clone using M13 primers. Purified PCR product (2 μg) was used as the template to synthesize the sense and antisense strands of *Mi-cpl-1* using T7 and Sp6 transcription kits (Ambion). Synthesis of dsRNA was done by mixing of two ssRNAs followed by incubation at 65°C for 10 min and 37°C for 30 min. Confirmation of dsRNA synthesis was done by running a 2 μl aliquot on 1% agarose gel. Subsequently, dsRNA of an unrelated gene, *gfp* (green fluorescent protein), cloned in pGEM-T vector was generated to be used as the negative control.

### *in vitro* RNAi of *Mi-cpl-1*

To trace the efficiency of dsRNA uptake by nematodes, FITC (Fluorescein isothiocyanate) was used. Freshly hatched *M. incognita* J2s were soaked in 1 mg ml^−1^ *Mi-cpl-1* dsRNA in a soaking buffer containing FITC (0.1 mg ml^−1^) and 50 mM octopamine, incubated for 6 h in dark on a rotator at room temperature. J2s incubated in soaking buffer alone and in soaking buffer with *gfp* dsRNA were used as the controls to demonstrate the target-specific silencing. After incubation, J2s were rinsed five times with sterile water by centrifugation. Treated nematodes were observed using a Zeiss Axiocam MRm fluorescence microscope to monitor the uptake efficiency.

To examine the *Mi-cpl-1* silencing effect on nematodes, attraction and migration assay was conducted on pluronic gel (Wang et al., 2009). Three ml of 23% pluronic gel F-127 (Sigma) evenly mixed with approximately 1000 dsRNA fed J2s was pipetted onto 35 × 10 mm Petri dish. Tomato (cv. Pusa Ruby) seedling with 1 cm long root tip was placed at the center of the dish and incubated at 27°C. Attraction of J2 toward root tip was monitored at 4 h and 8 h. After 24 h roots were stained with acid fuchsin to record the number of J2s that had invaded the root (Byrd et al., 1983). Three replicates were taken for each treatment and repeated at least twice. Data were subjected to One-Way ANOVA and CRD test followed by Duncan's multiple-comparison test with significance level at *P* < 0.05 using SAS software (version 9.3).

To analyse the echamber were inoculated with xpression of *Mi-cpl-1* gene in the dsRNA fed nematodes, total RNA was isolated from nematodes treated with dsRNA of *Mi-cpl-1*, dsRNA of *gfp* and soaking buffer alone. qRT-PCR of the synthesized cDNA was performed as described above. At least two biological and three technical replicates were used for each of the samples. Expression was calculated using 2^−ΔΔCT^, and student's *t*-test was performed.

### Preparation of RNAi construct of *Mi-cpl-1* for HIGS

The pHELLSGATE12 vector (RNAi Gateway ready) was obtained from Commonwealth Scientific and Industrial Research Organization (CSIRO), Australia (http://www.csiro.au/Organisation-Structure/Divisions/Plant-Industry/RNAi/Plantvectors.aspx) (Helliwell and Waterhouse, 2003). Partial sequence of *Mi-cpl-1* (366 bp) was initially amplified from pGEM-T clone with attB1 and attB2 sites at the upstream and downstream of the gene sequence, respectively, through PCR. Primer details are given in Table 1. AttB flanked PCR product of *Mi-cpl-1* was sub-cloned into the entry vector pDONR221, using BP clonase enzyme. *Mi-cpl-1* fragment was subsequently cloned into the binary vector pHELLSGATE12 in sense and antisense orientation separated by an intron, using recombination-based Gateway cloning technique, mediated by the LR clonase enzyme (Invitrogen). The recombinant clone was transformed to *E. coli* DH5α cells. To confirm the target gene orientation, colony PCR was carried out with four different sets of primers (gene-specific forward and reverse; CaMV 35S promoter forward and attB2 reverse; CaMV 35S terminator forward and attB2 reverse; *nptII* forward and reverse primers). To ensure the orientation of the insert, PCR products were sequenced.

Binary plasmid (pHELLSGATE12) containing the RNAi construct was mobilized to the competent cells of *Agrobacterium tumefaciens* strain GV3101 using freeze and thaw method (Jyothishwaran et al., 2007). Positive clones were selected through colony PCR and maintained in Yeast-Peptone-Agar medium with selection against the antibiotics rifampicin (25 μg ml^−1^), gentamicin (50 μg ml^−1^) and spectinomycin (50 μg ml^−1^).

### Co-transformation and selection of tomato explants

Tomato (cv. Pusa Ruby) seeds were surface sterilized with 70% ethanol for 5 min and 1% NaOCl for 2 h, followed by four times washing with sterile water. Seeds were germinated on MS agar (pH 5.8) medium. Leaf explants of 1 cm^2^ cut from the fortnight old tomato seedling were used for *Agrobacterium*-mediated transformation. Explants were plated on pre-cultivation medium (MS + 0.5 mg L^−1^ IAA + 0.5 mg L^−1^ Zeatin). After 3 days, pre-cultivated explants were infected with *A. tumefaciens* (GV3101) cells harboring the RNAi construct for 5 min. Agroinfected leaves were dried by soaking in sterile tissue paper and plated on co-cultivation media (MS + 0.5 mg L^−1^ IAA + 0.5 mg L^−1^ Zeatin). After 2 days, co-cultured leaf discs were transferred to the selection medium (MS + 0.5 mg L^−1^ IAA + 0.5 mg L^−1^ Zeatin + 100 mg L^−1^ kanamycin + 250 mg L^−1^ cefotaxime). Selection plates were incubated at 25°C with a 16/8 h light/dark cycle. After 25 days, shoots that emerged from the explants were excised and sub-cultured in the fresh selection medium at 15 days interval for further shoot elongation. For root initiation, elongated shoots were kept in the rooting media (1/2 MS + 0.5 mg L^−1^ NAA + 100 mg L^−1^ kanamycin + 250 mg L^−1^ cefotaxime). The established plants with hardened roots were transferred to the transgenic glasshouse facility of ICAR-IARI, New Delhi for further growth and T_0_ seeds were produced.

### DNA isolation and PCR confirmation of primary transgenic plants

Genomic DNA was isolated from the fresh leaves of all the T_0_ events using NucleoSpin Plant II DNA extraction kit (Macherey-Nagel) following the manufacturer's protocol. PCR confirmation of the transgene was done with different sets of primers (gene-specific forward and reverse; CaMV 35S promoter forward and attB2 reverse; CaMV 35S terminator forward and attB2 reverse; *nptII* forward and reverse primers) (Table 1).

### Southern blot for transgenic tomato lines

To ensure the T-DNA integration, 15 μg DNA of each PCR-positive tomato line was digested with 50 U BamHI (New England Biolabs) for 16 h at 37°C. Digested DNA was resolved on 0.8% agarose gel and transferred by capillary action onto a nitrocellulose membrane (BioRad Zeta probe) in 10X saline sodium citrate (SSC) buffer [1.5 M NaCl, 0.15 M sodium citrate (pH 7.0)]. Fragment of *Mi-cpl-1* gene (366 bp) labeled with [*α*-^32^P]-dCTP using mega prime DNA labeling kit (Amersham Pharmacia Biotech), was used as the probe. Probe was purified with Sephadex G-50 column (GE Healthcare Life Sciences) following manufacturer's instructions. Membrane was UV-crosslinked and hybridized overnight with radioactive probe in the hybridization buffer [2 M Na_2_HPO_4_ (pH 7.2), 10% sodium dodecyl sulfate (SDS), 0.5 M EDTA (pH 7.0)] at 65°C. Later, membrane was gradually washed three times in 3X SSC and 0.1% SDS; 0.5X SSC and 0.1% SDS followed by 0.1X SSC and 0.1% SDS, for 30 min each at 65°C (Southern, 1975), exposed to Fujifilm (Kodak) for 1–5 days at −80°C and developed. All experiments were conducted twice and showed similar results.

### Expression analysis of *Mi-cpl-1* in T_1_ transgenic plants

T_0_ seeds of tomato plant were germinated on MS media supplemented with 100 mg L^−1^ kanamycin and the seedlings were transferred to the medium containing ½MS, 0.25 mg L^−1^ GA_3_ and 100 mg L^−1^ kanamycin. Matured plants were subsequently transferred to 300 ml pots containing autoclaved soil and soil rite in 3:1 ratio. T_1_ plants were kept in a growth chamber at 28°C, 70% RH and 16: 8 h light: dark photoperiod. DNA was isolated from the fresh leaves of T_1_ plants as stated above and PCR confirmation of the transgene was done using different sets of primers (Table 1).

To analyse the transcript abundance of *Mi-cpl-1* gene in T_1_ plants, total RNA was isolated from fresh leaves of PCR positive events and reverse transcribed to cDNA. qRT-PCR analysis was performed as mentioned above. Average ΔCT values were obtained by calculating the difference between the Ct mean of *Mi-cpl-1* and *18S rRNA* gene. At least two biological and three technical replicates were taken for each of the samples. Primer details are given in Table 1.

### Northern blot for transgenic tomato lines

Total RNA and small RNA were isolated from the fresh leaves of PCR-positive events using Nucleospin miRNA isolation Kit (Macherey-Nagel). 10 μg total RNA of each sample was denatured by heating at 68°C for 10 min, separated on 2% high resolution MetaPhor agarose gel, and transferred to a nitrocellulose membrane as described above. Similarly, denatured small RNA was separated on 15% denaturing polyacrylamide gel and transferred to a membrane. Probes Mi-cpl-1 and U6 (small nuclear RNA as positive control) were synthesized by PCR using primers Mi-cpl-1F, Mi-cpl-1R, U6F, and U6R, respectively (Table 1), and radiolabeled with [*α*-^32^P]-dCTP by mega prime DNA labeling kit (Amersham Pharmacia Biotech). Membranes were UV-crosslinked and hybridized with the purified probes overnight at 42°C in hybridization buffer. Membranes were washed and X-ray films were developed as described above. All experiments were conducted twice and showed similar results.

### Bioefficacy studies of T_1_ plants expressing dsRNA of *Mi-cpl-1* against *M. incognita*

A fortnight old T_1_ plants kept in growth chamber were inoculated with approximately 500 freshly hatched J2s in the vicinity of the root zone, and incubated at 28°C, 70% relative humidity and 16: 8 h light: dark photoperiod. Plants were harvested 35 days post-inoculation (DPI), and roots were washed free of soil. Observations were taken on total number of galls, females, egg masses, and eggs per egg mass for each plant. In addition, to determine the effect of HIGS on *M. incognita* reproductive potential, nematode multiplication factor [(number of eggmasses × number of eggs per egg mass) ÷ nematode inoculum level] was calculated for each transgenic event. All the data were compared with the wild type plants inoculated with similar number of *M. incognita* J2s, grown under similar conditions. Infection assay was set up as randomized complete block design with six replicates per treatment. Data were subjected to One-Way ANOVA and CRD test followed by Duncan's multiple-comparison test with significance level at *P* < 0.05 using SAS software (version 9.3). Experiment was conducted twice and showed similar results.

### Expression analysis of *Mi-cpl-1* in nematodes extracted from transgenic plants

Mature females feeding on the roots of wild type and transgenic RNAi plants were isolated at 35 days post-infection stage by macerating the infected galled root tissue under the microscope using forceps. Extracted nematodes were frozen immediately in liquid N_2_ and stored at −80°C. Total RNA was isolated and reverse transcribed to cDNA as described above. Transcript accumulation of *Mi-cpl-1* was analyzed by qRT- PCR. Primer details are given in Table 1. Two biological and three technical replicates were used. Expression was calculated using 2^−ΔΔCT^, and student *t*-test was performed.

## Results

### Differential expression of *Mi-cpl-1* transcript in different stages of *M. incognita*

**Figure 2:**
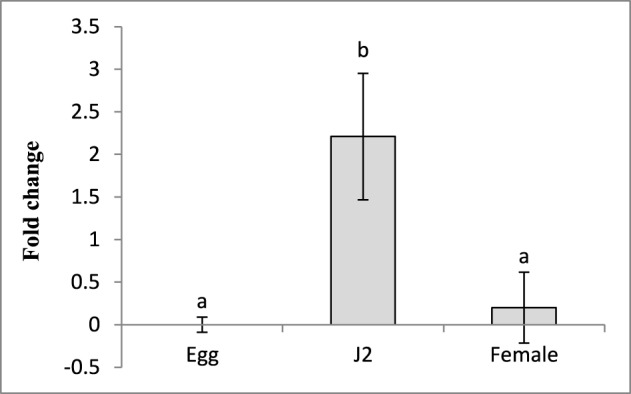
**Relative abundance of the transcript of *Mi-cpl-1* gene in different developmental stages of *M. incognita***. Using 2^-ΔΔCT^ method relative expression level was quantified. Using the transcript level of *Mi-cpl-1* in eggs as reference, expression of *Mi-cpl-1* was found to be significantly higher at J2 stage. Each bar represents the mean ±SE of *n* = 3, and bars with different letters denote a significant difference at *P* < 0.05, student's *t*-test.

Sequencing and BLAST analysis of the cloned fragment of *Mi-cpl-1* (366 bp) gene amplified from the cDNA of *M. incognita*, revealed 100% similarity to the reported coding sequence of *Mi-cpl-1*. To determine the transcription pattern of *Mi-cpl-1* throughout the nematode development process, stage-specific expression of *Mi-cpl-1* was investigated in eggs, J2s and mature females of *M. incognita*, using qRT-PCR. Using the expression level of *Mi-cpl-1* in eggs as reference, *Mi-cpl-1* was upregulated 2.2-fold in infective J2 in terms of fold change values, while no significant (*P* < 0.05) difference in expression between the eggs and females could be observed (Figure 2). Higher transcript accumulation of *Mi-cpl-1* in infective J2 indicates that, function of *Mi-cpl-1* is crucial at the early stage of nematode development, during which nematodes find the suitable host, penetrate the root and establish a compatible relationship with the host tissue, leading to formation of GC.

### *in vitro* RNAi of *Mi-cpl-1* in *M. incognita* J2

**Figure 3:**
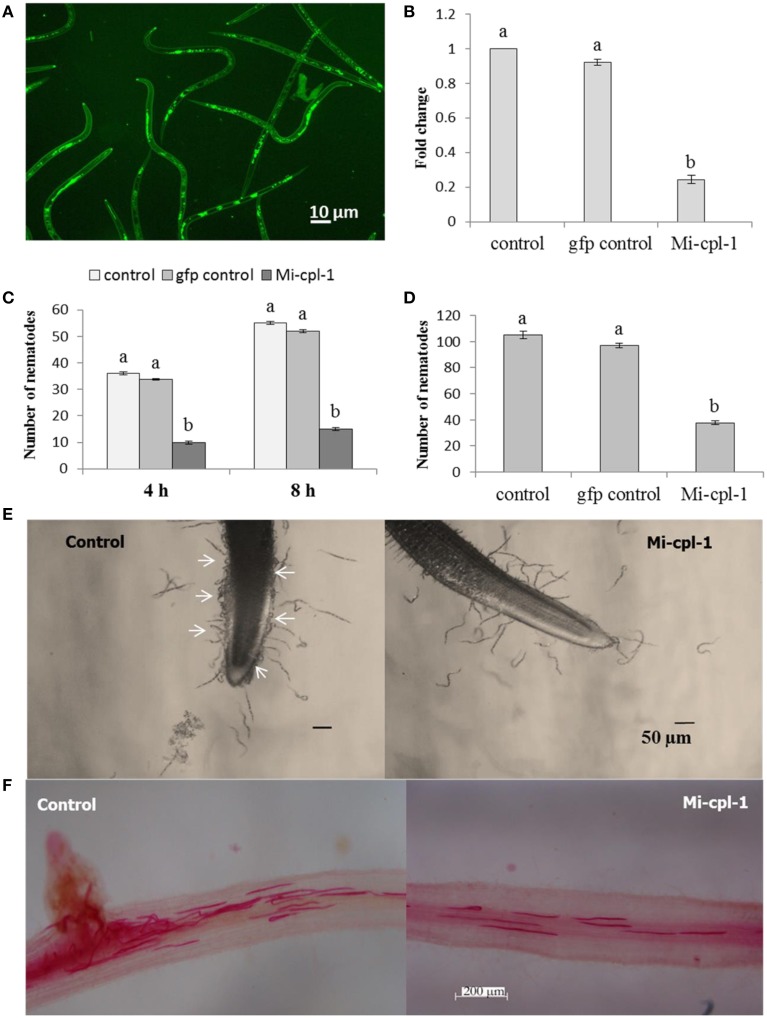
**Effect of *in vitro* RNAi of *Mi-cpl-1* in *M. incognita* J2. (A)** Fluorescence microscopy showing the ingestion of FITC in *M. incognita* J2 after 6 h treatment with octopamine (50 mM) in soaking buffer. **(B)** Real-time PCR analysis of *Mi-cpl-1* transcript abundance. Expression was quantified as fold change values calculated by 2^-ΔΔCT^ method. **(C)** Attraction of J2s to tomato root tip at different time points (4 h and 8 h) in pluronic gel. **(D)** Nematode penetration of tomato root after 24 h. **(E)** Attraction assay in pluronic gel. Arrows indicate control J2s were attracted in greater number toward tomato root tip compared to dsRNA fed J2s. **(F)** Invasion of *M. incognita* J2 inside tomato roots after 24 h. Tomato roots supported less number of dsRNA treated J2s compared to control (Scale bar = 200 μm). Nematodes were stained with acid fuchsin (Byrd et al., 1983). Control-treatment with soaking buffer alone; gfp control-treatment with soaking buffer containing 1 mg ml^−1^ *gfp* dsRNA; Mi-cpl-1-treatment with soaking buffer containing 1 mg ml^−1^ *Mi-cpl-1* dsRNA. Each bar represents the mean ±SE of *n* = 3, and bars with different letters denote a significant difference at *P* < 0.05.

To study the silencing of *Mi-cpl-1 in vitro, M. incognita* J2s were soaked in a buffer containing dsRNA of *Mi-cpl-1* gene. Fluorescence microscopic observations revealed that the J2s had efficiently ingested the FITC marker and likely also the dsRNA molecules (Figure 3A). According to qRT-PCR results, approximately 76% reduction in mRNA abundance was observed in *Mi-cpl-1* dsRNA fed J2s compared to J2s treated with soaking buffer alone or *gfp* dsRNA. There was no significant (*P* < 0.05) difference between the expression level of J2s treated with soaking buffer alone and *gfp* dsRNA (Figure 3B).

To further evaluate the effect of *Mi-cpl-1* RNAi, chemotactic behavior of J2s was assessed using attraction assay of dsRNA treated nematodes toward tomato root tip in pluronic gel (Wang et al., 2009). Silencing of *Mi-cpl-1* had negative impact on nematode chemotaxis as J2s treated with *Mi-cpl-1* dsRNA were attracted to tomato root tip in significantly (*P* < 0.05) lesser number than the control worms at 4 h and 8 h (Figures 3C,E). After 24 h penetration study was carried out, the results of which corroborated with the attraction experiment (Figures 3D,F).

These findings suggest that *Mi-cpl-1* RNAi effects are significant at the transcript level as soaking buffer alone or soaking buffer with *gfp* dsRNA did not affect the dsRNA phenotypes and *Mi-cpl-1* displayed no genetic redundancy.

### Molecular analysis of primary transgenic events

Recombinant pHELLSGATE12 containing dsRNA construct of *Mi-cpl-1* (Figure S1) was transformed into the tomato plants (Figure S2), and T_0_ plants were generated. To ensure the complete integration of T-DNA into the genome of *S. lycopersicum*, initial genotyping of T_0_ plants were carried out using PCR analysis. Presence of gene specific, sense, antisense and selectable marker fragment was detected in all the events (Figure S3).

**Figure 4:**
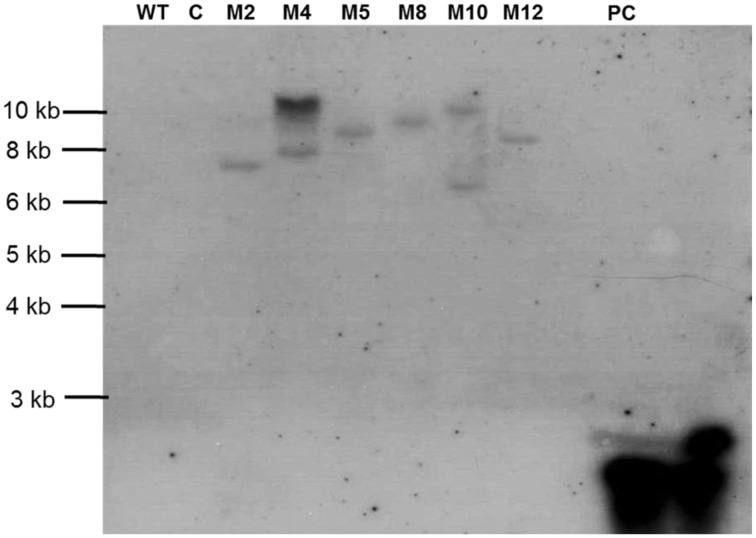
**Southern blot for transgenic tomato harboring dsRNA of *Mi-cpl-1***. Lines used include Tomato “Pusa Ruby” wild type (WT), transgenic empty pHELLSGATE12 vector control (C) and transgenic pHELLSGATE12-*Mi-cpl-1* (M2, M4, M5, M8, M10, M12). Probe Mi-cpl-1 (PC) was used for the hybridization.

To determine the T-DNA integration pattern in transgenic plants, genomic Southern analysis was performed with six PCR-confirmed events. Lines M4 and M10 had multiple insertions of the RNAi transgene, while M2, M5, M8, and M12 showed single copy integration pattern. No hybridization signal was detected in the wild type and empty vector control plants (Figure 4).

Based on the outcome of integration studies, four events carrying the hairpin construct of *Mi-cpl-1* (M2, M5, M8, and M12) were subjected to expression and bioefficacy analysis in the successive generation. T_1_progeny plants were generated in the growth chamber by germinating the seeds of T_0_plants in the presence of antibiotic kanamycin. T_1_plants were genotyped by PCR and expected fragments were detected, which indicates the stable integration and inheritance of RNAi transgene in the progeny plants.

### Expression analysis of *Mi-cpl-1* in T_1_ transgenic events

**Figure 5:**
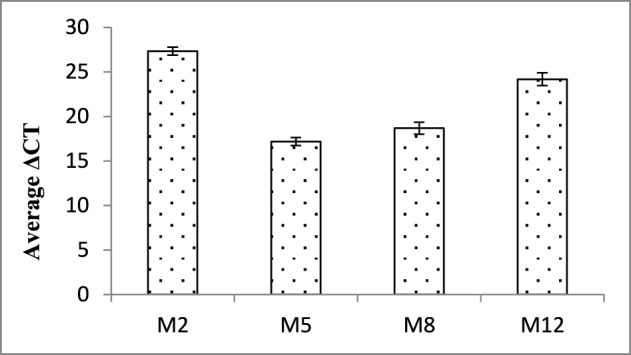
**Expression analysis of *Mi-cpl-1* gene in T_1_ plants**. ΔCT values were calculated using the difference in the Ct mean of *Mi-cpl-1* and *18S rRNA* gene. Each bar represents the mean ±SE of *n* = 3.

In order to validate the expression of dsRNA transcript and its abundance, qRT-PCR analysis of the selected events (M2, M5, M8, and M12) was carried out. An increased transcript abundance of *Mi-cpl-1* was recorded in all the events, and among them event M2 had the highest expression level in terms of average ΔCT values (Figure 5).

**Figure 6:**
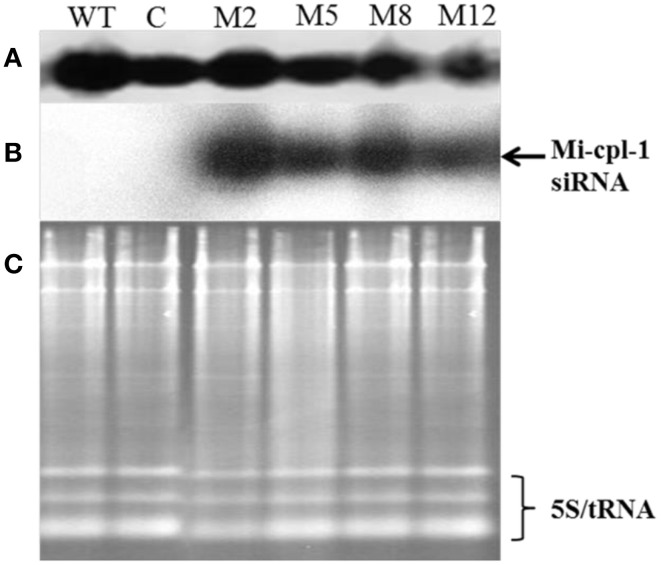
**Production of *Mi-cpl-1*-specific small RNAs in transgenic tomato plants.(A)** Northern blot for U6 small nuclear RNA as control, **(B)** Northern blot showing *Mi-cpl-1* siRNAs hybridizing with probe Mi-cpl-1, and **(C)** Agarose gel showing total RNA loading control in Tomato “Pusa Ruby” wild type (WT), transgenic empty pHELLSGATE12 vector control (C), and transgenic pHELLSGATE12-*Mi-cpl-1* (M2, M5, M8, M12) plants.

As a key component of HIGS, expression of siRNA of *Mi-cpl-1* was demonstrated by northern analysis in four transgenic events (M2, M5, M8, and M12). As expected, presence of *Mi-cpl-1* siRNA could not be detected in the wild type and empty vector control plants (Figure 6).

### Evaluation of T_1_ plants for resistance against *M. incognita*

**Figure 7:**
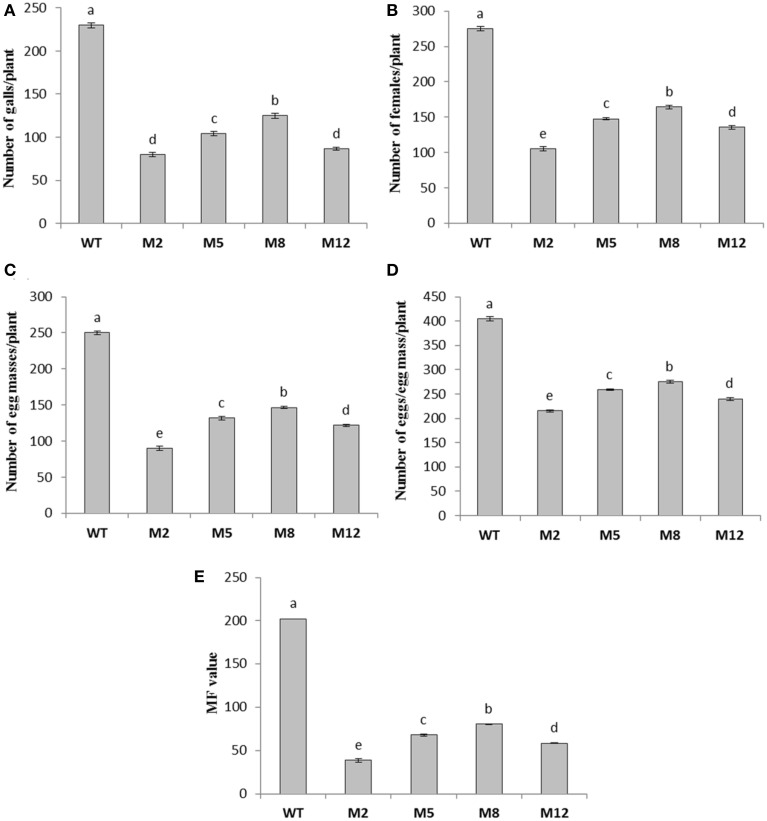
**Effect of host induced gene silencing of *Mi-cpl-1* on development and reproduction of *M. incognita***. Relative number of galls **(A)**, females **(B)**, egg masses **(C)**, eggs/egg mass **(D)** and the respective multiplication factor (MF) of *M. incognita* **(E)** in different transgenic events (M2, M5, M8, and M12) and wild type plants (WT) at 35 DPI. Each bar represents the mean ±SE of *n* = 4, and bars with different letters denote a significant difference at *P* < 0.05, CRD test.

**Figure 8:**
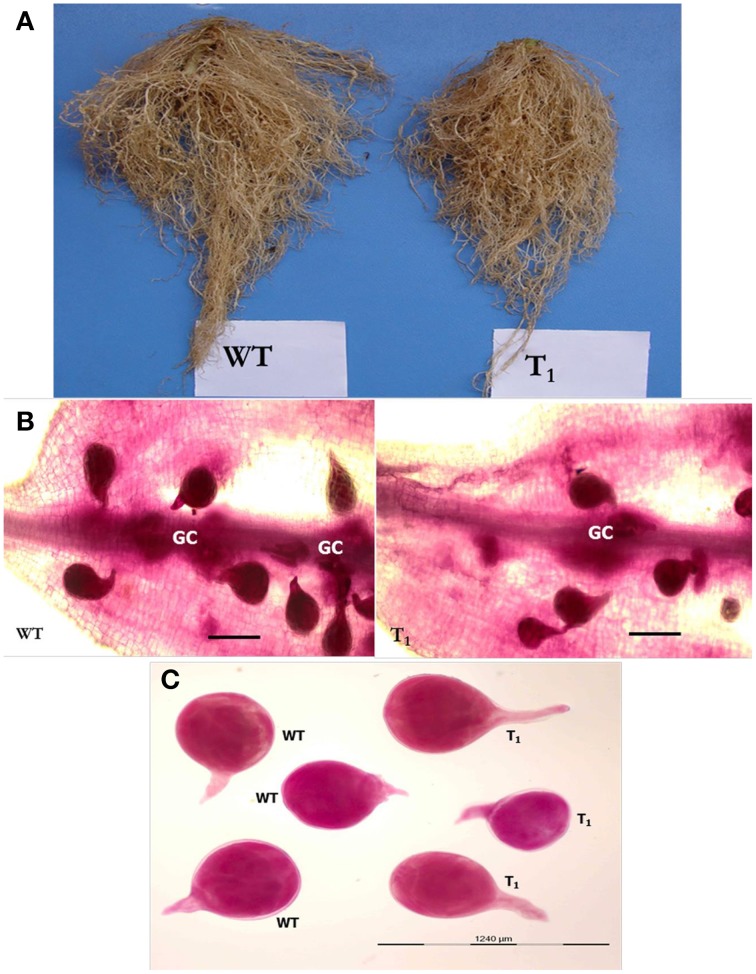
**Comparison of *M. incognita* infection in transgenic (T_1_) and wild type (WT) tomato plants at 35 DPI. (A)** Intensity of galling was comparatively higher in control plants than the transgenic plants. **(B)** Stained females of *M. incognita* inside the tomato root. Transgenic plants had supported less number of females compared to wild type plants. GC—giant cell (Scale bar = 500 μm). **(C)** Size and shape of mature females of *M. incognita* isolated from tomato roots. Nematodes were stained with acid fuchsin (Byrd et al., 1983).

To assess whether *in planta* expression of *Mi-cpl-1* dsRNA exhibits resistance to *M. incognita*, four T_1_ tomato lines (M2, M5, M8, and M12) harboring the RNAi construct were inoculated with 500 *M. incognita* J2s per plant. In comparison to wild type plants no apparent phenotypical variation was observed in transgenic plants. At 35 DPI, the average number of galls per plant was reduced significantly (*P* < 0.05) by 45.6–65.2% in RNAi lines M8, M5, M12, and M2 compared to the wild type (Figure 7, Table 2). Accordingly, there was a reduction in number of females and the percentage reduction ranged between 40.2–61.8% in RNAi lines compared to the wild type (Figure 7, Table 2). Post-parasitic juveniles of *M. incognita* become exposed to the target dsRNA while they ingest the plant cell contents during GC formation. Dynamic maintenance of GC entails the development of the juvenile into an egg-laying female and no establishment of GC may lead to nematode death or to development as the non-feeding male. Therefore, plant-mediated silencing of *Mi-cpl-1* gene resulted in reduced root galling due to impeded developmental progression of *M. incognita* and eventually, lesser number of developed females was observed in the transgenic plants compared to the wild type plants (Figures 8A,B). However, mature females extracted from both transgenic and wild type plant were similar in shape and size (Figure 8C). Comparable results were obtained when the fecundity of nematodes was measured in RNAi lines as number of egg masses and number of eggs per egg mass, with significant reductions of 41.3–64% and 31.9–46.8% (*P* < 0.05) compared to the wild type, respectively (Figure 7, Table 2). Last but not least, the nematode multiplication factor (MF) which determines the reproductive fitness and parasitic success of a given nematode was significantly (*P* < 0.05) reduced by 60–80.8% in RNAi lines compared to the wild type plants (Figure 7, Table 2). In essence, host-delivered RNAi of *Mi-cpl-1* gene confers resistance to *M. incognita* by inducing negative effect on nematode infection, development and reproduction.

**Table 2:** **Percentage reduction in different parameters of nematode development and reproduction on transgenics (M2, M5, M8, and M12) compared to the wild type plants**.

	**M2**	**M5**	**M8**	**M12**
Number of galls	65.21	54.63	45.65	62.31
Number of females	61.81	46.30	40.24	50.66
Number of egg masses	64	47.33	41.33	51.33
Number of eggs/egg mass	46.82	36.10	31.98	40.64
MF value	80.83	66.33	60.09	71.11

**Figure 9:**
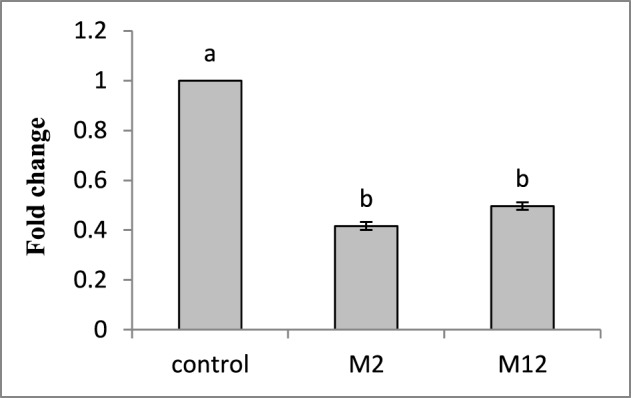
**Transcript levels of *Mi-cpl-1* gene in *M. incognita* females that developed in the dsRNA expressing transgenic tomato lines**. Using *18S rRNA* gene as reference, expression was quantified as fold change values calculated by 2^-ΔΔCT^ method. Each bar represents the mean ±SE of *n* = 3, and bars with different letters denote a significant difference at *P* < 0.05, student's *t*-test.

To investigate the effect of HIGS on the target gene abundance in nematodes, qRT-PCR was performed to analyse the possible change in transcript level of *Mi-cpl-1* in *M. incognita* females harvested from T_1_transgenic lines (M2 and M12) at 35 DPI. Using 18S ribosomal DNA as reference, it was found that the expression level of *Mi-cpl-1* in *M. incognita* females that developed on RNAi lines M2 and M12 was reduced significantly (*P* < 0.05) by 59 and 51%, respectively, compared to females extracted from wild type plants (Figure 9).

## Discussion

The parasitic success of *M. incognita* largely depends on the exploitation of its arsenal of parasitism genes to circumvent the plant immune responses and to induce and maintain the GC in the host vascular tissue. The current study was devised to downregulate an essential gene of *M. incognita* using HIGS approach and to assess the knock-down effect on nematode biology. According to the available data, stable transformation of agronomically important crops to enhance the RNAi-mediated resistance against nematodes has been accomplished in tobacco (*Nicotiana tabacum* L.), soybean [*Glycine max* (L.) Merr.] and potato (*S. tuberosum* L.), with varying degree of success (Steeves et al., 2006; Yadav et al., 2006; Fairbairn et al., 2007; Klink et al., 2009; Ibrahim et al., 2011; Papolu et al., 2013; Dinh et al., 2014). The targeted nematode genes in some of these reports are conserved in other animals, and due to their potential off-target effects the engineered crops may prove to be ineligible for field trial. Cathepsin L-like cysteine proteinases are an attractive group of candidate genes for RNAi-induced downregulation, not only because they are crucial for parasitic aspects of plant-nematode interaction, but they lack significant homology to genes in other organisms. In an earlier report, tobacco transgenic lines containing the dsRNA (201 bp) and fusion (600 bp) construct of *Mi-cpl-1* gene had been generated, although nematodes feeding on those transgenics did not affect the gall formation or egg-mass production by females (Antonino de Souza Júnior et al., 2013). As reviewed by Sharp (2001), long dsRNA sequences may lead to greater RNAi effect compared to short dsRNAs. Conversely, longer dsRNA molecule (>400 bp) causes less effective silencing as they may not readily be assimilated in the nematode body (Dalzell et al., 2009). Therefore, in the present study, stable tomato transgenic lines overexpressing a 366 bp dsRNA construct of *Mi-cpl-1* gene have been developed to elucidate the role of this gene in plant-nematode interaction. To the best of our knowledge, this is the first detailed investigation to address the function of *Mi-cpl-1* gene in *M. incognita*-*S. lycopersicum* interaction using both *in vitro* and *in vivo* RNAi strategy.

Initially, to investigate the differential expression of *Mi-cpl-1* transcripts throughout the developmental stage of *M. incognita*, qRT-PCR analysis was used. The transcript level of *Mi-cpl-1* was relatively higher in pre-parasitic J2 than females and eggs. This finding is in agreement with the RT-PCR data reported by Neveu et al. (2003). Shingles et al. (2007) demonstrated the similar expression of *Mi-cpl-1* in pre-parasitic J2, young and mature female of *M. incognita*, and slightly lower expression in older females, based on RT-PCR data. According to Antonino de Souza Júnior et al. (2013), transcript abundance of *Mi-cpl-1* was comparatively higher in eggs and parasitic juveniles than females and pre-parasitic J2 of *M. incognita*, as observed by qRT-PCR. These discrepancies might have resulted from the sequence polymorphisms between populations of *M. incognita*. Using *in situ* hybridization assay, activity of this enzyme was detected in the intestinal cells of pre-parasitic J2 (Neveu et al., 2003) and females (Shingles et al., 2007). Combining all these stage-specific expression data, it can be speculated that *M. incognita* development (J2 to J3 to J4 to female) is largely dependent on the smooth functioning of this digestive enzyme. Developmental stages of *M. incognita* occur in close interaction with the host root tissues, indicating *Mi-cpl-1* is indeed related to the parasitic phase of plant-nematode interaction.

*In vitro* RNAi can be used for functional validation of specific nematode genes and to assess their suitability for HIGS approach. Using this strategy, efficient suppression of the target genes with corresponding aberrant phenotypes have been successfully documented in *M. incognita* (Rosso et al., 2005; Huang et al., 2006; Dubreuil et al., 2007; Shingles et al., 2007; Papolu et al., 2013; Dong et al., 2014), *M. javanica* (Adam et al., 2008; Gleason et al., 2008), *G. pallida* (Urwin et al., 2002; Kimber et al., 2007), *G. rostochiensis* (Chen et al., 2005), *H. glycines* (Urwin et al., 2002; Lilley et al., 2005; Bakhetia et al., 2007), *H. schachtii* (Vanholme et al., 2007), *Pratylenchus* spp. (Joseph et al., 2012; Tan et al., 2013), *Bursaphelenchus xylophilus* (Park et al., 2008; Cheng et al., 2010) etc. Therefore, in the present investigation, *Mi-cpl-1* gene was silenced *in vitro* to analyse its function in nematode behavior. qRT-PCR results revealed that silencing of *Mi-cpl-1* gene leads to 76% transcript knock-down in the dsRNA treated worms compared to the control J2s. Target-specific downregulation of *Mi-cpl-1* gene was also confirmed due to the absence of RNAi effect in the *gfp* dsRNA treated worms. To determine the phenotypic changes in nematodes induced by *in vitro* RNAi, chemotactic behavior of J2s toward tomato root tip was studied. In compliance with the expression data, dsRNA treated *M. incognita* J2s were attracted to and invaded the tomato root in lesser number compared to control worms. Hence, reduction in infection ability of J2 could be attributed to the nutritional deficiency experienced by the nematodes due to depletion in Mi-cpl-1 enzyme.

Furthermore, tomato plants were transformed to express the *Mi-cpl-1* dsRNA and challenge inoculated with *M. incognita* J2 to delineate the function of *Mi-cpl-1* in nematodes through HIGS. Substantial reduction in infection, development and reproduction of *M. incognita* was observed in the transgenic lines compared to the wild type plants. Nematode multiplication factor which determines its successful establishment in the host plants was reduced significantly in the transgenic lines. No apparent morphological variation was observed in tomato lines compared to the wild type plants, indicating the focused RNAi effect. In addition, qRT-PCR analysis revealed the higher expression of transgenes in RNAi lines which commensurate with the bioefficacy data. Finally, the detection of target gene siRNA in the tomato plants using northern analysis provided the ultimate evidence for plant-mediated RNAi of *Mi-cpl-1* gene that minimized the nematode virulence and reproduction. Therefore, *in planta* reduction of *Mi-cpl-1* transcripts diminished the parasitic success of *M. incognita* in tomato. Silencing of a nematode effector gene (*16D10*) has been reported by Huang et al. (2006) to lower the number of galls and egg masses by 63–90% and 69–93%, respectively, in Arabidopsis plants infected with *M. incognita, M. javanica, M. arenaria* or *M. hapla*. This classical report has been regarded as the commencement of HIGS strategy targeting plant-parasitic nematodes. Since then, several nematode genes have been targeted to generate the hairpin expressing transgenic plants with variable levels of resistance against phytoparasitic nematodes (Dutta et al., 2015), and the level and range of resistance conferred by tomato lines to *M. incognita* in the current study is comparable with those reports.

In order to evaluate the long-term effects of RNAi on nematode development, females of *M. incognita* that developed on the plants expressing *Mi-cpl-1* dsRNA were extracted and subjected to qRT-PCR analysis. A substantial reduction in *Mi-cpl-1* expression was recorded in females extracted from RNAi plants and consequently, the nematodes that fed on the transgenic plants were less successful in further development. It can be hypothesized that the silencing effect of *Mi-cpl-1* in *M. incognita* is systemic and propagates upon the uptake of host-derived dsRNAs/siRNAs through stylet to the entire body. RNAi effect is transmitted as the nematode subsequently molts to the female stage and ultimately the offspring having defunct *Mi-cpl-1* gene maybe produced. The heritable nature of RNAi was previously described in *C. elegans* (Grishok et al., 2000), and may occur in plant nematodes in similar fashion (Fairbairn et al., 2007; Sindhu et al., 2009; Papolu et al., 2013; Dinh et al., 2014). It remains inconclusive whether *M. incognita* ingested the host-derived dsRNAs and processed them into siRNAs using Dicer enzyme or (if) they directly took up the plant-processed siRNAs in the current study. Either or both may be possible as root-knot nematodes are known to efficiently ingest relatively large biomolecules (Urwin et al., 1997; Li et al., 2007; Zhang et al., 2012).

Although the transgenic delivery of dsRNA/siRNA of *Mi-cpl-1* gene via plant cells to *M. incognita* J2 elicited developmental retardation in the feeding nematodes, none of the tomato lines exhibited complete resistance in the current study. The reason could be explained by the fact that nematode genes may have additive, combinatorial or redundant function in the plant-nematode interface. Therefore, to achieve complete and durable nematode resistance more than one nematode gene should be targeted for simultaneous interference. In this direction, effectiveness of combinatorial RNAi was investigated in *H. glycines* (Bakhetia et al., 2008) and *M. incognita* (Antonino de Souza Júnior et al., 2013). In either case, however, desired results could not be obtained perhaps because high level of dsRNA delivery saturates the dsRNA processing ability of nematodes. Nevertheless, crossing of different RNAi lines resulting in the co-expression of compound dsRNA constructs in the same transgenic plant enhances the efficacy of RNAi (Huang et al., 2006; Charlton et al., 2010). This strategy ensures the continuous delivery of bioactive RNA species to the feeding nematodes.

Classical breeding approaches are time-consuming and additional impediments including linkage drag, compatibility barrier, and gene loss during introgression are associated with it (Hermsen, 1994). Biosafety-related issues associated with the transgenic crops expressing nematicidal proteins, such as cystatins or cry proteins have not been addressed yet. In view of this, RNAi-based transgenics have been a viable option due to the lack of impact of RNAi on non-target species and no transgenic protein is produced in RNAi plants (Atkinson et al., 2009). The RNAi-based genetic engineering strategy presented here could provide an attractive alternative to breed root-knot nematode-resistant tomatoes. The biosafety level of this novel strategy can be improved by using tissue-specific promoters that can efficiently deliver the nematode dsRNAs into the active feeding cells. To date, no crop varieties with resistance to *Meloidogyne* spp. have yet been bioengineered for field trial owing to the lack of awareness of the damage potential of phytonematodes among the plant breeders (Atkinson et al., 2012). Given that how destructive nematodes can be (Jones et al., 2013), it is imperative that pyramiding of nematode genes with the other pathogen genes in the same crop plant may provide unprecedented broad-spectrum resistance to various pests and pathogens. To ensure the global food security, future studies should focus on the evaluation of RNAi technology in staple crops such as cereals and pulses to manage the plant nematodes and to explore the suitability of this technology at the field level.

## Author contributions

Conceived and designed the experiments: TD, UR, AS. Performed the experiments: TD, PP, PB, DC. Analyzed the data: TD. Wrote the manuscript: TD.

### Conflict of interest statement

The authors declare that the research was conducted in the absence of any commercial or financial relationships that could be construed as a potential conflict of interest.
