# Supplementary material for: Tomato transgenic plants expressing hairpin construct of a nematode protease gene conferred enhanced resistance to root-knot nematodes
Source: Front Microbiol. 2015 Apr 1;6:260. doi: 10.3389/fmicb.2015.00260 (PMC4381642; doi:10.3389/fmicb.2015.00260)
Supplement: Supplementary file 1 [file DataSheet1.DOCX]

**SUPPORTING FIGURES**

**Figure S1. Development of RNAi construct of *Mi-cpl-1* for *in planta* validation.** **A.** Schematic representation of the modular binary destination vector pHELLSGATE12. The T-DNA portion contained ccd B gene flanked by attR1 and attR2 site in one gateway cassette and ccd B gene flanked by reverse complement of attR1 and attR2 site in another gateway cassette, separated by an intron under the control of promoter CaMV 35S. **B.** Procedure for RNAi vector construction. Recombination between attL1/attL2 site of entry clone (pDONR221) and attR1/attR2 site of destination vector (pHELLSGATE12), mediated by LR clonase enzyme yields the recombinant vector containing RNAi construct. When the construct is expressed in plants, a hairpin RNA (hpRNA) with the intron spliced out is produced to silence the target gene of the invading nematodes.

**Figure S2.** **Transformation of tomato plants with RNAi constructs and generation of transgenic lines (T_0_). A.** 15 day old tomato seedlings, **B.** Pre-cultivation of leaf discs, **C.** Co-cultivation with *Agrobacterium*, **D.** Selection plate containing kanamycin, **E.** Callus initiation of the explants, **F.** Callus differention, **G.** Shoot induction in the explant, **H.** Root induction in the explant.

**Figure S3. PCR confirmation of *Mi-cpl-1* gene in tomato transgenic lines (T_0_). A.** Amplification of the target gene using gene specific primers (366 bp). **B.** Amplification of sense strand using primers 35S promoter forward and attB2 reverse (501 bp). **C.** Amplification of the antisense strand using primers 35S terminator forward and attB2 reverse (465 bp). **D.** Amplification of nptII gene (750 bp). Lanes - 1: 100 bp DNA Ladder, 2-13: T_0_ events.


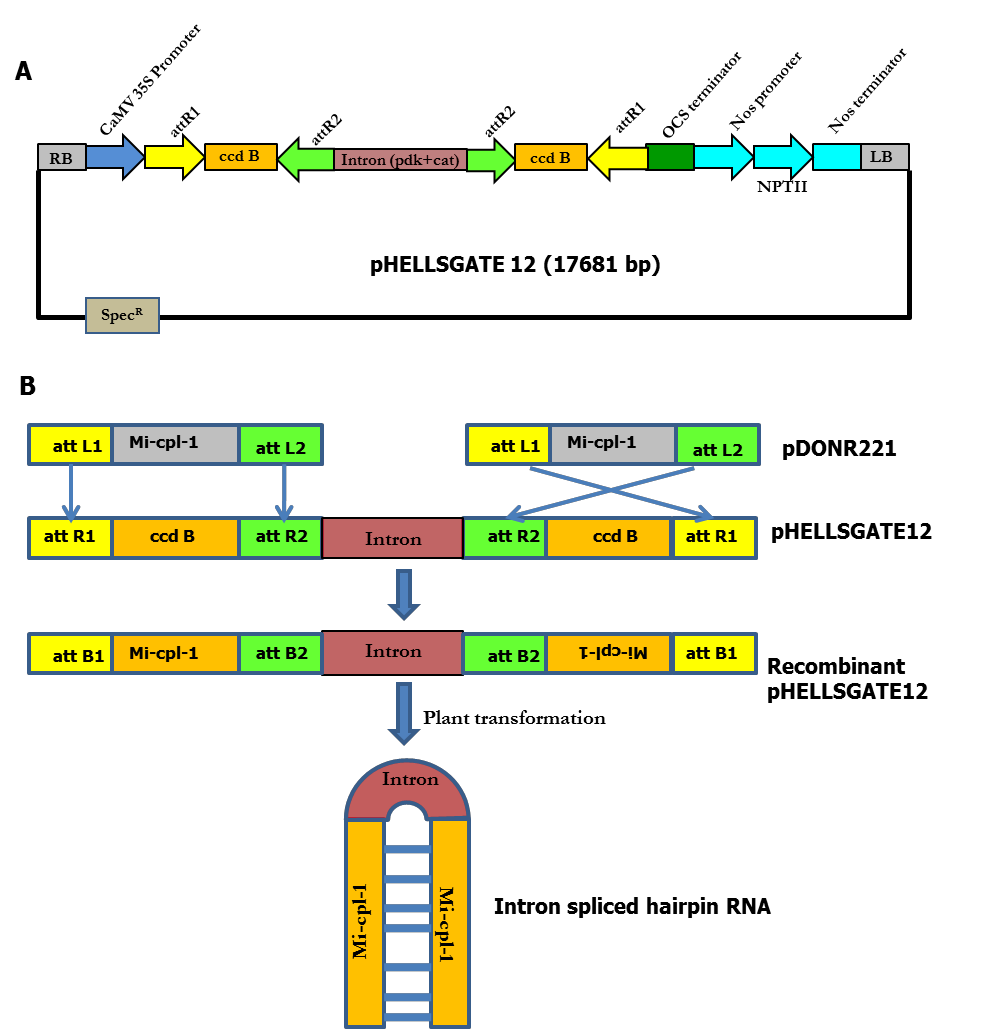


**Figure S1.** Development of RNAi construct of *Mi-cpl-1* for *in planta* validation. **A.** Schematic representation of the modular binary destination vector pHELLSGATE12. The T-DNA portion contained ccd B gene flanked by attR1 and attR2 site in one gateway cassette and ccd B gene flanked by reverse complement of attR1 and attR2 site in another gateway cassette, separated by an intron under the control of promoter CaMV 35S. **B.** Procedure for RNAi vector construction. Recombination between attL1/attL2 site of entry clone (pDONR221) and attR1/attR2 site of destination vector (pHELLSGATE12), mediated by LR clonase enzyme yields the recombinant vector containing RNAi construct. When the construct is expressed in plants, a hairpin RNA (hpRNA) with the intron spliced out is produced to silence the target gene of the invading nematodes. (600×600dpi)

| 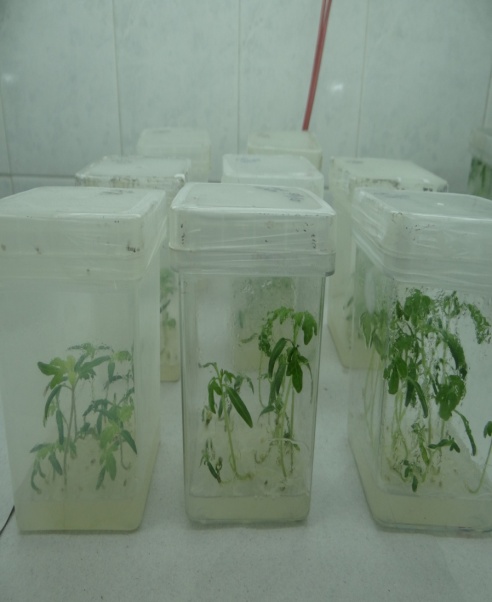  **A** | 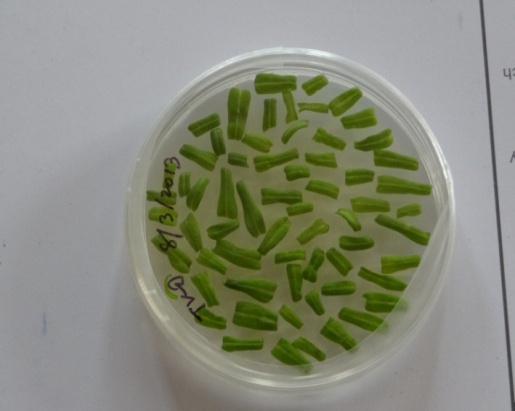  **B** | 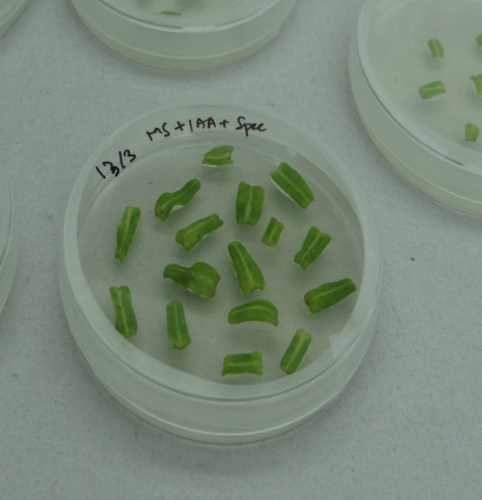  **C** | 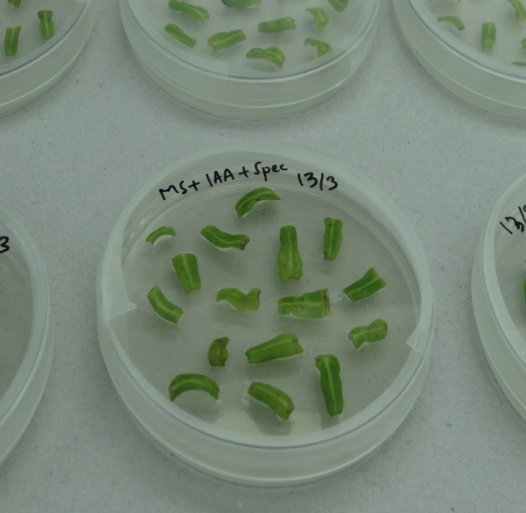  **D** |
| --- | --- | --- | --- |
| 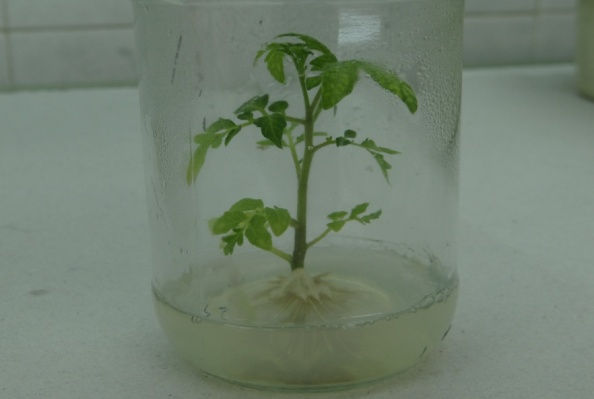  **H** | 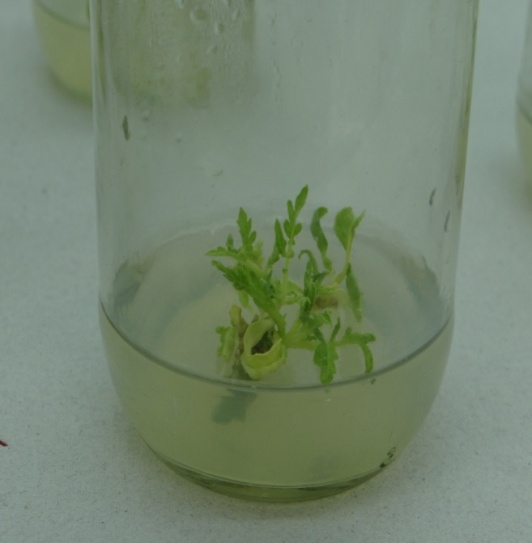  **G** | 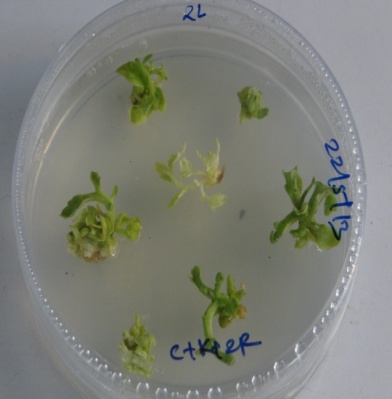  **F** | 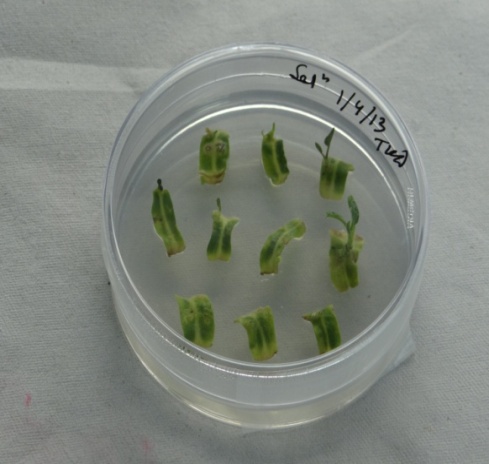  **E** |

**Figure S2.** Transformation of tomato plants with RNAi constructs and generation of transgenic lines (T_0_). **A.** 15 day old tomato seedlings, **B.** Pre-cultivation of leaf discs, **C.** Co-cultivation with *Agrobacterium*, **D.** Selection plate containing kanamycin, **E.** Callus initiation of the explants, **F.** Callus differention, **G.** Shoot induction in the explant, **H.** Root induction in the explant.


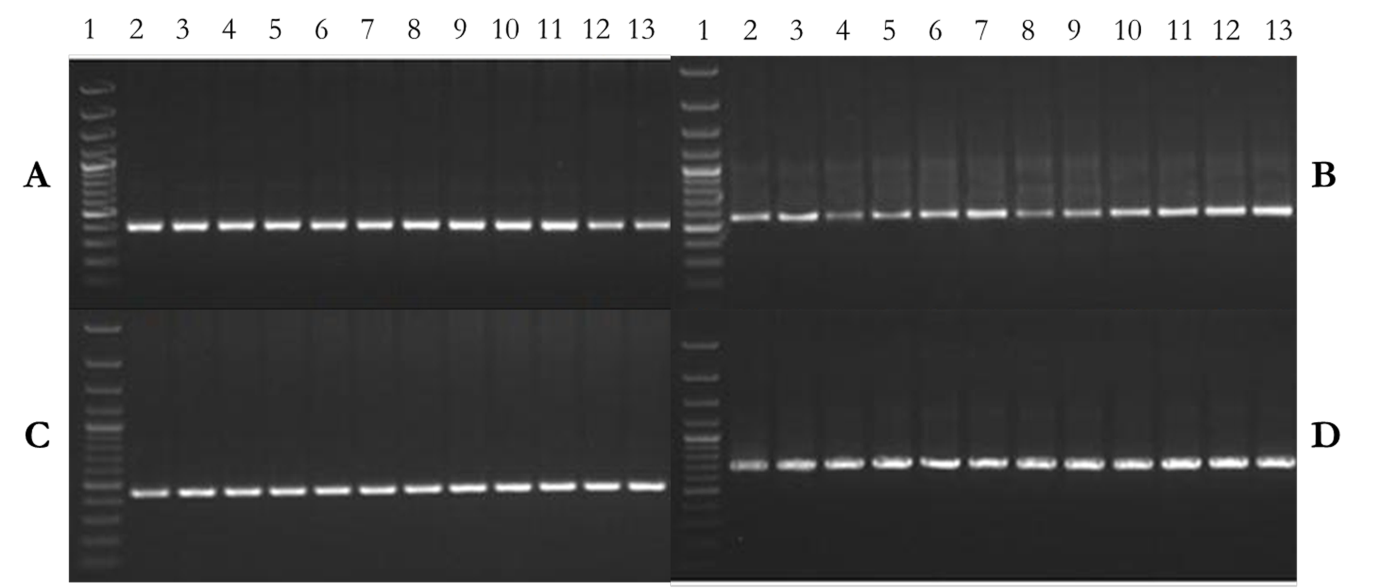


**Figure S3. PCR** confirmation of *Mi-cpl-1* gene in tomato transgenic lines (T_0_). **A.** Amplification of the target gene using gene specific primers (366 bp). **B.** Amplification of sense strand using primers 35S promoter forward and attB2 reverse (501 bp). **C.** Amplification of the antisense strand using primers 35S terminator forward and attB2 reverse (465 bp). **D.** Amplification of nptII gene (750 bp). Lanes - 1: 100 bp DNA Ladder, 2-13: T_0_ events. (300×300dpi)
